# Lipoprotein a Combined with Fibrinogen as an Independent Predictor of Long-Term Prognosis in Patients with Acute Coronary Syndrome: A Multi-Center Retrospective Study

**DOI:** 10.3390/jcdd9100322

**Published:** 2022-09-23

**Authors:** Cai-Yan Cui, Tao Ye, Lian-Chao Cheng, Lin Tong, Lan Tong, Zhen Zhang, Lin Cai

**Affiliations:** Department of Cardiology, The Affiliated Hospital of Southwest Jiaotong University, The Third People’s Hospital of Chengdu, Cardiovascular Disease Research Institute of Chengdu, 82 Qinglong St., Chengdu 610031, China

**Keywords:** lipoprotein a, fibrinogen, acute coronary syndrome, long-term prognosis, predictor

## Abstract

Background: Patients with acute coronary syndrome (ACS) still have a high risk of recurrence of major adverse cardiovascular and cerebrovascular events (MACCE). However, there are rare studies on the prediction of MACCE in patients with ACS using lipoprotein a [Lp(a)] combined with fibrinogen. The aim of this study was to analyze the predictive value of Lp(a) combined with fibrinogen for the long-term prognosis of patients with ACS. Methods: 804 patients with ACS admitted to 11 tertiary general hospitals in Chengdu from January 2017 to June 2019 were included in the study. According to the Lp(a) 300 mg/L, patients were assigned to the non-high Lp(a) group and high Lp(a) group. Patients were assigned to the non-high or high fibrinogen groups using the fibrinogen level of 3.08 g/L. Subsequently, patients were divided into group A, B, or C by Lp(a) combined with fibrinogen. The study endpoints were MACCE, including all-cause death, non-fatal myocardial infarction, non-fatal stroke, and revascularization. The incidences of MACCE among groups were compared. Lp(a), fibrinogen, Lp(a) combined with fibrinogen classifications were each added into the basic model to construct three new models. The C-index, net reclassification index (NRI) and integrated discrimination improvement (IDI) of the three new models were then compared. **Results**: The median follow-up was 16 months. During follow-up, the cumulative incidence of MACCE in group C was significantly higher than that measured in group A and B (*p* < 0.001). The results of the multivariate Cox regression analysis of MACCE showed that Lp(a) ≥300 mg/L with fibrinogen ≥3.08 g/L was an independent predictor of MACCE. According to the GRACE score and the statistical analyses, the basic model was constructed, which had a C-index of 0.694. The C-index, NRI, and IDI of the new model constructed using the basic model + Lp(a) combined with fibrinogen classification were 0.736, 0.095, and 0.094 respectively. Conclusions: Single Lp(a), single fibrinogen and Lp(a) combined with fibrinogen were independent predictors of MACCE in patients with ACS. The predictive value of Lp(a) combined with fibrinogen in patients with ACS was better than that of single Lp(a) and single fibrinogen.

## 1. Introduction

Coronary heart disease (CHD) is the most common type of cardiovascular disease [1]. Acute coronary syndrome (ACS) is an acute clinical type of CHD, that includes ST-segment elevation myocardial infarction (STEMI), non-ST-segment elevation myocardial infarction (NSTEMI), and unstable angina (UA) [1]. The prevalence of ACS in the world is still high. For example, in 2016, the number of patients with ACS in the United States was about 1.045 million, including 1.022 million cases of myocardial infarction and 23,000 cases of UA [2]. Mortality remains high in patients with ACS worldwide. According to the CRUSADE study, the in-hospital mortality of acute myocardial infarction (AMI) patients was 7%, 1-year mortality was 24%, 5-year mortality was 51%, and 8-year mortality was 65% in elderly patients (>65 yr) [3]. Data from the Healthcare and Utilization Project (HCUP) showed that 40% of patients with ACS had multiple readmissions, with more than 70% of patients were readmitted within 2 months of their first discharge [4]. The average cost of the first admission was $71,336 and the average cost of readmission was $53,290, with an average length of hospital stay of 5.4 days. Therefore, the high morbidity and mortality of ACS has seriously endangered the health of residents, causing huge social and economic burden, and has become a major global public health problem [5].

There are still some problems and dilemmas regarding the treatment of patients with ACS. In recent years, the development and application of percutaneous coronary intervention (PCI), coronary artery bypass grafting (CABG) and other reperfusion measures, as well as the establishment of chest pain centers has resulted in efficient treatment and improved prognosis of ACS patients [6]. However, in spite of standardized diagnosis and treatment as required by guidelines, some ACS patients still have a high risk of recurrence of major adverse cardiovascular and cerebrovascular events (MACCE), such as myocardial infarction, revascularization, stroke, and cardiogenic death [7]. How to identify ACS patients prone to MACCE and initiate active treatment measures is crucial to further improve the prognosis of ACS patients. 

Both lipoprotein a [Lp(a)] [8,9,10] and fibrinogen [11,12] can promote atherosclerosis and thrombosis. The results of several studies have suggested that Lp(a) [13,14] and fibrinogen [15] were independent predictors of MACCE in ACS patients, although their predictive effect is limited. We hypothesized that the combination of Lp(a) and fibrinogen may improve the ability to predict the risk of MACCE in ACS patients. Although there have been many reports on the predictive potential of these two blood tests, research on the combined use of Lp(a) and fibrinogen is lacking. The current study therefore aimed to investigate the predictive value of Lp(a) combined with fibrinogen for assessing the risk of MACCE in ACS patients. 

## 2. Materials and Methods

### 2.1. Study Population

**Figure 1:**
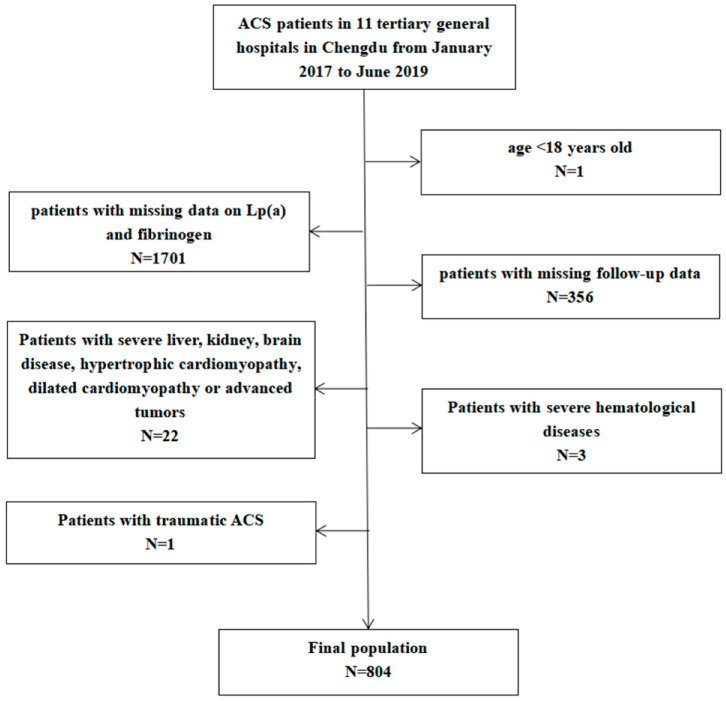
Screening process for patients. ACS: acute coronary syndrome.

The study was a multicenter retrospective study of ACS patients who visited 11 tertiary general hospitals in Chengdu from January 2017 to June 2019. Inclusion criteria: patients who met the diagnosis of STEMI, NSTEMI or UA [1]. Exclusion criteria: (1) age <18 years old; (2) patients with missing data on Lp(a) and fibrinogen; (3) patients with missing follow-up data; (4) severe liver, kidney, brain disease, hypertrophic cardiomyopathy, Patients with dilated cardiomyopathy or advanced tumors; (5) severe hematological diseases; (6) traumatic ACS. The screening process for patients is shown in Figure 1.

The study was approved by the medical ethics committee of the Third People’s Hospital of Chengdu and its clinical trial registration number is ChiCTR1900025138.

According to the guidelines and previous studies [16,17,18], patients with a Lp(a) level <300 mg/L were classified into the non-high Lp(a) group, and patients with a Lp(a) ≥300 mg/L were classified into the high Lp(a) group. According to previous studies, the cutoff value for fibrinogen grouping was a median of 3.08 g/L. Patients with a fibrinogen level <3.08 g/L were classified into the non-high fibrinogen group, and patients with a fibrinogen level ≥3.08 g/L were classified into the high fibrinogen group. The patients were then divided into group A, B, and C according to Lp(a) combined with fibrinogen. Group A: Lp(a) <300 mg/L and fibrinogen <3.08 g/L; group B: Lp(a) ≥300 mg/L and fibrinogen <3.08 g/L or Lp(a) <300 mg/L and fibrinogen ≥3.08 g/L; group C: Lp(a) ≥300 mg/L and fibrinogen ≥3.08 g/L.

Baseline data were collected using the electronic medical record system of the hospitals and follow-up data by telephone and during an outpatient clinic appointment. Baseline data included demographic and medical history, clinical symptoms and signs, laboratory tests, whether or not the patient had received reperfusion therapy and its method. Follow-up data were collected from the date of admission of the patient with the information obtained from the medical record systems of the hospitals. Follow-up information after discharge was collected at outpatient clinic visits, by telephone calls or from readmission records in the medical record system. Follow-up data mainly included death and its causes, myocardial infarction, revascularization, stroke and its type, and the time of occurrence of the above events.

The end point was the occurrence of MACCE during the follow-up. MACCE is a composite event of all-cause death, nonfatal myocardial infarction, nonfatal stroke, and revascularization.

### 2.2. Definitions

Active smoking was defined as continuous smoking for more than 6 months or currently smoking; smoking cessation was defined as past smoking but not currently smoking [19]. Diabetes mellitus (DM) was diagnosed as either a fasting plasma glucose level ≥7.0 mmol/L, 2 h plasma glucose level ≥11.1 mmol/L, or previously diagnosed with DM or current use of hypoglycemic therapy [20]. Hypertension was diagnosed by either blood pressure measured three times on different days, with a systolic blood pressure ≥140 mmHg or diastolic blood pressure ≥90 mmHg, or a previous diagnosis of hypertension, or current use of antihypertensive treatment [21]. Coronary multivessel disease was defined as two or more vessels with ≥75% coronary stenosis. Left main or anterior descending coronary artery disease was diagnosed as left main or anterior descending coronary artery stenosis ≥75%. 

### 2.3. Statistical Analysis

Statistical analysis was performed using SPSS 20.0 software. Categorical variables were expressed as number (percentage) with comparisons between groups performed by the chi-square test or Fisher’s exact test. Continuous variables were expressed as the mean ± standard deviation or median (interquartile range) and compared, as appropriate, using the ANOVA or Kruskal–Wallis H test. Survival curves were estimated and plotted by the Kaplan–Meier, with the log-rank test used to compare the survival curves of two or more groups. Cox regression analysis was used to assess whether Lp(a), fibrinogen, and Lp(a) combined with fibrinogen classifications were associated with worse long-term prognosis. The parameters included in the multivariate survival analyses were age, gender, smoking, ACS classification, and history of coronary heart disease or cerebral infarction. Factors included in the multivariate Cox regression analysis were those with a *p* value < 0.1 in the univariate Cox regression analysis and Lp(a), fibrinogen, and Lp(a) combined with fibrinogen classifications. A basic model was constructed and the Lp(a), fibrinogen, and Lp(a) combined with fibrinogen classifications then added to the basic model to establish three new prediction models for the occurrence of MACCE. The C-statistic of the models was calculated by R language 4.0.3 software. The net reclassification index (NRI) and integrated discriminant improvement index (IDI) of the three new models were calculated with reference to the basic model. All the statistical tests were two-tailed, with a *p* value < 0.05 considered to be statistically significant.

## 3. Results

### 3.1. Baseline Characteristics of the Study Population

A total of 804 ACS patients treated in 11 tertiary general hospitals in Chengdu from January 2017 to June 2019 were included in the study. According to the grouping of Lp(a) >300 mg/L, there were 642 cases in the non-high Lp(a) group and 162 cases in the high Lp(a) group. According to the median fibrinogen of 3.08 g/L, there were 407 cases in the non-high fibrinogen group and 397 cases in the high fibrinogen group. Then there were 345 cases in group A, 359 cases in group B, and 100 cases in group C. The average age was 66 ± 13 years and 593 cases (73.8%) were male. The median follow-up time was 16 months (9 months, 24 months). The incidence of MACCE was 17.5%. 

The comparison of the clinical baseline characteristics of the three groups is shown in Table 1. Compared with patients in group A and B, patients in group C had a higher proportion of STEMI, hypertension and previous renal dysfunction, and faster heart rate, higher triglyceride levels, and a higher proportion of receiving PCI treatment (all *p* < 0.05).

### 3.2. Comparison of Cumulative Incidence of MACCE

#### 3.2.1. Comparison of the Cumulative Incidence of MACCE between Non-High-Lp(a) Group and High-Lp(a) Group

**Figure 2:**
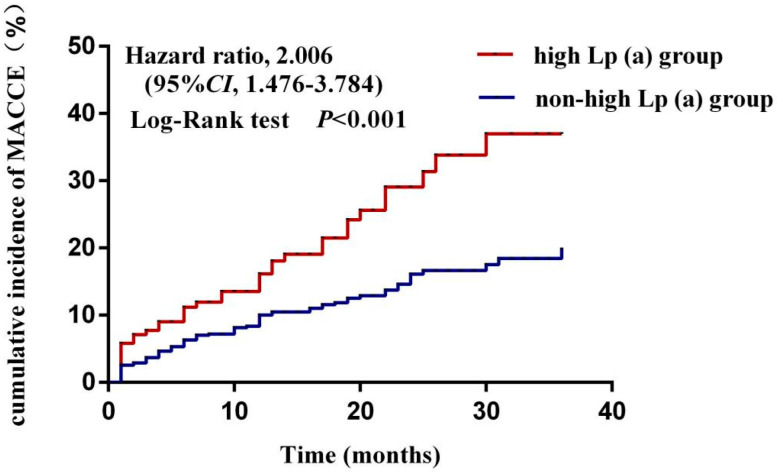
Comparison of the cumulative incidence of MACCE between high-Lp(a) group and non-high-Lp(a) group.

During follow-up, the cumulative incidence of MACCE was significantly higher in the high Lp(a) group than in the non-high Lp(a) group [HR 2.006, 95%CI (1.476–3.784), *p* < 0.001] (Figure 2). 

#### 3.2.2. Comparison of the Cumulative Incidence of MACCE between Non-High-Fibrinogen Group and High-Fibrinogen Group

**Figure 3:**
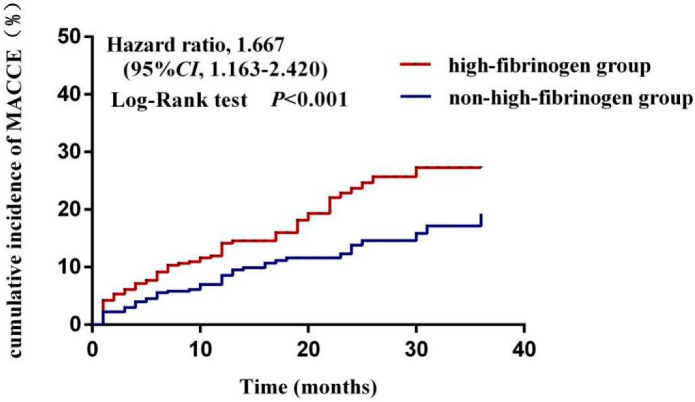
Comparison of the cumulative incidence of MACCE between non-high-fibrinogen group and high-fibrinogen group.

During follow-up, the cumulative incidence of MACCE was higher in the high-fibrinogen group than in the non-high-fibrinogen group [HR 1.667, 95%CI (1.163–2.420), *p* < 0.001] (Figure 3).

#### 3.2.3. Comparison of the Cumulative MACCE Incidence between Groups of Lp(a) Combined with Fibrinogen

**Figure 4:**
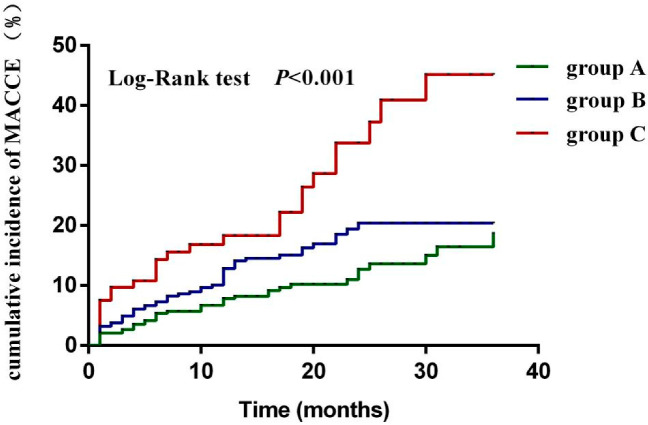
Comparison of the cumulative MACCE incidence between groups of Lp(a) combined with fibrinogen.

During follow-up, the cumulative incidence of MACCE was significantly higher in group C [Lp(a) ≥ 300 mg/L and fibrinogen ≥ 3.08 g/L] than in group A and B (*p* < 0.001) (Figure 4). 

### 3.3. Multivariate Cox Regression Analysis of MACCE in Patients with ACS

Variables with a *p* < 0.1 in the univariate Cox regression analysis of MACCE were included in the multivariate Cox regression analysis, with the results were shown in Table 2. Age [HR 1.044, 95%CI (1.023–1.066), *p* < 0.001], white blood cell count [HR 1.102, 95%CI (1.043–1.164), *p* = 0.001], coronary multivessel disease [HR 2.156, 95%CI (1.400–3.321), *p* < 0.001] were independent predictors of MACCE in patients with ACS.

On the basis of the above multivariate Cox regression analysis, Lp(a) classification, fibrinogen classification, Lp(a) combined with fibrinogen classification were added to perform the multivariate Cox regression analysis of MACCE. The results are shown in Table 3. Lp(a) ≥300 mg/L [HR 2.193, 95%CI (1.400–3.436), *p* = 0.001], fibrinogen ≥3.08 g/L [HR 1.814, 95%CI (1.120–2.937), *p* = 0.015], Lp(a) ≥300 mg/L and fibrinogen ≥3.08 g/L [HR 3.388, 95%CI (1.816–6.321), *p* < 0.001] were independent predictors of MACCE in ACS patients.

### 3.4. The Construction of MACCE Risk Forecast Models

According to the “GRACE score”, systolic blood pressure, age, heart rate, and serum creatinine were included, and the white blood cells, coronary artery multi-vessel lesions (*p* < 0.05 in the multivariate Cox regression analysis of MACCE) were also included to construct the basic model. Lp(a) classification, fibrinogen classification and Lp(a) combined with fibrinogen classification were added to the basic model respectively to construct three new models. The C-Statistic, NRI, and IDI of the three new models are shown in Table 4.

## 4. Discussion

In previous studies, it remained controversial whether or not Lp(a) predicted prognosis in ACS patients. A previous study included patients with CHD who received PCI and divided them into high Lp(a) group and low Lp(a) group, and then divided them further into two subgroups: high low density lipoprotein cholesterol (LDL-C) group and low LDL-C group. The results showed that the cumulative incidence of major adverse cardiovascular events (MACE) in the high Lp(a) group was significantly higher than that in the low Lp(a) group in the high LDL-C subgroup, while there was no difference in the low LDL-C subgroup. This study concluded that when LDL-C was controlled below the target value, Lp(a) was not a risk factor for MACE in patients with CHD [22]. Accordingly, some scholars have proposed that when Lp(a) levels are elevated, the most appropriate treatment is LDL-C-lowering therapy rather than Lp(a). However, recent studies have taken a different view of this situation. For example, the results of the JUPITER study [23] showed that in the rosuvastatin group, elevated Lp(a) was an independent predictor of MACE in patients with CHD. However, recent findings have suggested that elevated Lp(a) are not independent predictor of MACCE in ACS patients [24]. The above studies suggest that the predictive value of Lp(a) for the prognosis of ACS patients remains controversial. In this regard, our study used a Lp(a) level of 300 mg/L to stratify patients according to the recommendations of the guidelines and the method used in previous studies. The results showed that Lp(a) ≥300 mg/L was an independent predictor of MACCE in ACS patients [HR 2.006, 95%CI (1.476–3.784), *p* < 0.001].

In this study, Lp(a) ≥300 mg/L combined with fibrinogen ≥3.08 g/L was an independent predictor of MACCE in ACS patients during follow-up [HR 3.388, 95%CI (1.816–6.321), *p* < 0.001]. It also had a higher risk ratio compared to a single high level of Lp(a) or a single high level of fibrinogen (3.388 vs. 2.193 vs. 1.814). In the MACCE risk forecast models of ACS patients, the C-statistic of basic model, basic model + Lp(a) classification, basic model + fibrinogen classification, and basic model + Lp(a) combined with fibrinogen classification were: 0.694, 0.723, 0.721, 0.736 respectively. These results indicated that basic model +Lp(a) combined with fibrinogen classification had higher discrimination and better predictive effect. Meanwhile, compared with the basic model, the NRI of the basic model +Lp(a) combined with fibrinogen classification was 0.095, indicating that 9.5% of patients in the basic model could be classified correctly using this combined model. The predictive potential of the basic model +Lp(a) combined with fibrinogen classification was also higher than that of the basic model + Lp(a) classification (5.7%) and basic model + fibrinogen classification (7.4%). Compared with the basic model, the IDI of basic model +Lp(a) combined fibrinogen classification was 0.094, indicating that the prediction probability of basic model +Lp(a) combined fibrinogen classification increased by 9.4%, which was higher than that of basic model +Lp(a) classification (6.7%) and basic model + fibrinogen classification (4.0%). In conclusion, the ability to predict MACCE in ACS patients using Lp(a) combined with fibrinogen is superior to either single Lp(a) or single fibrinogen. The reasons may be as follows: (1) It can be seen from the statistical analyses of our study that the prevalence of hypertension was higher in ACS patients with elevated Lp(a) and fibrinogen. Hypertension can promote the formation and development of atherosclerosis by increasing the concentration of angiotensin II [25], resulting in activation of sympathetic nerves [26], increased the mechanical pressure of blood vessels [27], and activation of coagulation factors [28]. De et al. [29] also showed that hypertension was an independent risk factor for recurrent myocardial infarction, revascularization, and all-cause mortality in STEMI patients. (2) This study observed that ACS patients with elevated Lp(a) and elevated fibrinogen had a higher proportion of previous renal dysfunction, higher serum creatinine, and lower eGFR. ACS patients with renal insufficiency may have both platelet dysfunction and coagulation dysfunction, with a dual risk of bleeding and ischemia/thrombosis [30]. Clinical studies have shown that renal insufficiency is a risk factor for poor prognosis in patients with ACS [31,32]. 

This study had certain limitations: (1) The research objective of the study was to investigate ACS patients and whether the study results are applicable to stable CHD and other patient populations need to be confirmed by other studies; (2) the study was a retrospective design with some bias; (3) in the study, both Lp(a) and fibrinogen levels were measured at admission and were not monitored during follow-up. Changes in Lp(a) and fibrinogen levels from admission to follow-up may have clinical significance; (4) the methods used to measure Lp(a) and fibrinogen levels were different in the various hospitals and therefore there were systematic errors in the data; (5) the sample size in our study was not big enough, thus the patients with Lp(a) ≥300 mg/L and fibrinogen <3.08 g/L were combined with patients with Lp(a) <300 mg/L and fibrinogen ≥3.08 g/L as group B.

## 5. Conclusions

The results of our study showed that elevated Lp(a) combined with elevated fibrinogen was an independent predictor for the occurrence of MACCE in patients with ACS, and the predictive ability was better than that of a single elevated Lp(a) and a single elevated fibrinogen, which was conducive to risk stratification of ACS patients and further targeted diagnosis and treatment measures. It provides a new concept and possibility to improve the quality-of-life and prognosis of ACS patients.

## Figures and Tables

**Table 1:** Baseline characteristics.

	Group A (*n* = 345)	Group B (*n* = 359)	Group C (*n* = 100)	Statistic	***p***-Value
Demographic					
Age ($\overset{-}{x}$ *± s*, years)	65 ± 13	66 ± 14	66 ± 14	1.453	0.235
Male [*n*(*%*)]	258 (74.8)	258 (71.9)	77 (77)	1.394	0.498
Medical history					
Smoking [*n*(*%*)]				4.921	0.295
Never smoking	175 (50.7)	176 (49.2)	38 (38.4)		
Quit smoking	40 (11.6)	40 (11.2)	14 (14.1)		
Continue smoking	130 (37.7)	142 (39.7)	47 (47.5)		
Drinking history [*n*(*%*)]				0.178	0.996
Never drinking	242 (70.6)	251 (70.5)	68 (69.4)		
Quit drinking	34 (9.9)	33 (9.3)	10 (10.2)		
Continue drinking	67 (19.5)	72 (20.2)	20 (20.4)		
Hypertension [*n*(%)]	191 (44.6)	232 (35.4)	47 (47.0)	8.068	0.018
Diabetes [*n*(*%*)]	83 (24.1)	106 (29.5)	24 (24.0)	3.066	0.216
Coronary heart disease [*n*(%)]	66 (19.1)	58 (16.2)	17 (17.0)	1.066	0.587
Abnormal kidney function [*n*(%)]	4 (1.25)	16 (4.5)	6 (6.1)	9.061	0.011
ACS [*n*(%)]				32.628	<0.001
UA	86 (24.9)	66 (18.4)	9 (9.0)		
NSTEMI	92 (26.7)	102 (28.4)	12 (12.0)		
STEMI	167 (48.4)	191 (53.2)	79 (79.0)		
Clinical characteristic					
Clinical signs [*n*(*%*)]					
Chest pain	322 (95.3)	340 (95.0)	96 (97.0)	0.631	0.729
Nausea and vomiting	20 (5.9)	32 (9.0)	5 (5.1)	3.307	0.191
Profuse sweating	75 (22.2)	77 (21.8)	26 (26.3)	0.936	0.626
Dyspnea	10 (3.0)	18 (5.1)	3 (3.0)	2.313	0.315
syncope	9 (2.7)	10 (2.8)	1 (1.0)	1.077	0.584
$\mathrm{Systolic}\;\mathrm{blood}\;(\bar{x}$*± s*, mmHg)	132 ± 25	134 ± 25	132 ± 25	3.744	0.024
Heart rate [*M*(*QR*)]	85 (69,110)	80 (71,91)	90 (73,102)	8.467	0.015
Laboratory values					
BNP [*M*(*QR*), ng/L]	145.12 (33.15,465.37)	341.70 (133.10,711.64)	165.6 (79.80,2192.80)	27.677	<0.001
Serum creatinine [*M*(*QR*), μmol/L]	76.40 (62.50,90.00)	86.30 (66.95,112.30)	92.70 (65.10,176.83)	4.988	0.083
eGFR [*M*(*QR*), mL/min/1.73 m^2^]	92.4 (67.8,117.8)	82.7 (60.8,106.0)	82.6 (62.0,106.8)	0.617	0.734
Triglyceride [*M(QR)*, mmol/L]	1.12 (0.84,2.38)	1.44 (1.16,2.26)	1.61 (1.26,2.27)	7.758	0.021
Total cholesterol [*M(QR)*, mmol/L]	3.95 (3.32,4.50)	4.49 (3.84,5.13)	4.46 (2.88,5.56)	3.700	0.157
LDL-C [*M*(*QR*), mmol/L]	2.29 (1.47,3.08)	2.71 (2.17,3.28)	2.75 (2.24,3.68)	4.751	0.093
HDL-C [*M*(*QR*), mmol/L]	1.19 (0.95,1.51)	1.14 (0.93,1.22)	1.17 (0.83,1.37)	1.841	0.398
CRP [*M*(*QR*), mg/L]	4.60(2.65,6.30)	10.90 (3.35,43.24)	10.97 (1.68,34.60)	49.007	<0.001
Hemoglobin [*M*(*QR*),g/L]	135 (120,145)	135 (119,146)	134 (100,141)	10.714	0.005
Blood glucose [*M*(*QR*), mmol/L]	6.81 (5.79,9.76)	6.84 (5.77,8.90)	6.60 (5.08,8.35)	2.824	0.244
PCI [*n*(%)]	224 (64.9)	256 (71.3)	78 (78.0)	7.349	0.025
Multiple coronary arteries lesion [*n*(*%*)]	82 (33.7)	109 (38.2)	42 (47.7)	5.411	0.067
Left main or anterior descending lesion [*n*(%)]	123 (56.9)	158 (61.5)	52 (64.2)	1.668	0.434
Drug [*n*(%)]					
Dual antiplatelet drugs	266 (90.8)	296 (93.1)	83 (94.3)	3.798	0.434
Lipid-lowering drugs	304 (94.1)	328 (97.9)	85 (93.4)	7.149	0.028
β-blockers	231 (72.2)	241 (71.9)	60 (64.9)	1.471	0.479
ACEI/ARB	181 (57.6)	172 (52.0)	38 (46.9)	6.237	0.044

ACS: acute coronary syndrome; UA: unstable angina; STEMI: ST-segment elevation myocardial infarction; NSTEMI: non-ST-segment elevation myocardial infarction; BNP: brain natriuretic polypeptide; eGFR: estimated glomerular filtration rate; LDL-C: low density lipoprotein cholesterol; HDL-C: high-density lipoprotein cholesterol CRP: C-reactive protein; ACEI: angiotensin-converting enzyme inhibitors; ARB: angiotensin receptor blockers.

**Table 2:** Multivariate Cox regression analysis of MACCE in patients with ACS.

Variable	*β* Value	*p* Value	HR	95%CI	
Gender (Male vs. Female)	0.301	0.242	1.351	0.816	2.234
Age	0.043	<0.001	1.044	1.023	1.066
Systolic blood pressure	−0.009	0.075	0.991	0.982	1.001
Heart rate	0.008	0.141	1.008	0.997	1.018
Serum creatinine	0.002	0.417	0.998	1.002	1.003
Blood glucose	0.012	0.663	1.012	0.959	1.068
White blood cells	0.097	0.001	1.102	1.043	1.164
Hematocrit	−0.010	0.677	0.990	0.947	1.036
Multiple coronary artery lesions	0.768	<0.001	2.156	1.400	3.321

**Table 3:** Multivariate Cox regression analysis of MACCE in patients with ACS.

Variable	β Value	*p* Value	HR	95%CI	
Lp(a) ≥ 300 mg/L	0.785	0.001	2.193	1.400	3.436
Fibrinogen ≥ 3.08 g/L	0.596	0.015	1.814	1.120	2.937
Lp(a) combined with fibrinogen		0.001			
Group A	—	—	—	—	—
Group B	0.612	0.029	1.844	1.064	3.195
Group C	1.220	<0.001	3.388	1.816	6.321

**Table 4:** The comparison between four MACCE risk forecast model.

Model.	MACCE		
	C-Statistic (95%CI)	NRI	IDI
Basic model	0.694 (0.664–0.725)	—	—
Basic model + Lp(a) classification	0.723 (0.694–0.752)	0.057	0.067
Basic model + fibrinogen classification	0.721 (0.691–0.751)	0.074	0.040
Basic model + Lp(a) combined fibrinogen classification	0.736 (0.707–0.766)	0.095	0.094

## Data Availability

Data can be acquired on reasonable request from the correspondence.
